# Spatially Resolved Metabolomic Profiling Reveals Progression-Associated Metabolic Reprogramming in Colorectal Liver Metastasis

**DOI:** 10.3390/metabo16050293

**Published:** 2026-04-24

**Authors:** Ying Zhu, Yixuan Cai, Qianyu Wang, Hanchuan Guo, Qianqian Xie, Yingshi Xiang, Songlin Yu, Bin Wu, Ling Qiu

**Affiliations:** 1 Department of Laboratory Medicine, Peking Union Medical College Hospital, Chinese Academy of Medical Sciences & Peking Union Medical College, Beijing 100730, China; zhuying34@pumch.cn (Y.Z.); b2025001265@student.pumc.edu.cn (Y.C.); b2024001074@student.pumc.edu.cn (Q.X.); yusonglin@pumch.cn (S.Y.); 2 Department of General Surgery, Peking Union Medical College Hospital, Chinese Academy of Medical Sciences & Peking Union Medical College, Beijing 100730, China; b2023001210@pumc.edu.cn (Q.W.); s2024001110@student.pumc.edu.cn (H.G.); s2025001118@student.pumc.edu.cn (Y.X.); 3 State Key Laboratory of Complex Severe and Rare Diseases, Peking Union Medical College Hospital, Chinese Academy of Medical Sciences & Peking Union Medical College, Beijing 100730, China

**Keywords:** spatially resolved metabolomics, mass spectrometry imaging, colorectal cancer, liver metastasis, metabolic landscape

## Abstract

**Highlights:**

**What are the main findings?**

- Spatially resolved metabolomics revealed distinct metabolic heterogeneity across normal mucosa, primary tumors, and liver metastases in colorectal cancer.
- Lipid metabolism, particularly of glycerides and acylcarnitines, showed progression-associated alterations in colorectal liver metastasis.

**What are the implications of the main findings?**

- Spatial metabolomic profiling is an effective strategy for characterizing tumor heterogeneity and metabolic reprogramming during cancer progression.

**Abstract:**

**Background/Objectives**: Colorectal cancer (CRC) is a leading cause of cancer-related mortality, with colorectal liver metastasis (CRLM) being the major determinant of poor prognosis. Tumor metabolic reprogramming and spatial heterogeneity complicate biomarker discovery and clinical management. This study aimed to characterize the spatial metabolomic landscape of CRC and identify progression-associated metabolic alterations and potential metabolic signatures for liver metastasis. **Methods**: A total of 23 tissue samples were collected from patients with CRC, with and without liver metastasis. Air flow-assisted desorption electrospray ionization mass spectrometry imaging (AFADESI-MSI) was used to map the spatial metabolite distributions. Region-of-interest analysis guided by histopathology enabled comparative metabolomic profiling across different tissue types. Multivariate statistical analysis, pathway enrichment, and receiver operating characteristic (ROC) curve analyses were performed to identify key metabolic alterations and evaluate potential biomarker performance. **Results**: Distinct spatial metabolomic profiles were observed across normal mucosa, primary tumors, liver metastases, and normal liver tissues. In primary colorectal tumors, amino acid, purine, and choline metabolism were significantly upregulated, whereas liver metastases were characterized by elevated levels of triglycerides, diglycerides, cholesteryl esters, and acylcarnitines, indicating enhanced lipid synthesis, incomplete fatty acid oxidation, and/or mitochondrial dysfunction. Progression-associated analyses across tissue types revealed consistently increasing trends in glycerides and acylcarnitines, along with heterogeneous alterations in amino acids and phospholipids. Furthermore, 122 differential metabolites were identified between metastatic and non-metastatic CRC, and a four-lipid panel demonstrated strong discriminatory performance. **Conclusions**: This study provides a spatially resolved characterization of metabolic reprogramming during CRC progression and liver metastasis, highlighting lipid and amino acid metabolism as key features and revealing the metabolic signatures of CRLM.

## 1. Introduction

Colorectal cancer (CRC) is the third-most common cancer and the second-most common cause of cancer-related deaths worldwide [1]. Metastatic colorectal cancer (mCRC) is one of the most challenging clinical conditions and is characterized by high recurrence rates and poor prognosis [2]. More than 50% of patients with CRC experience cancer metastasis in their lifespan [3], and approximately 15–25% of newly diagnosed patients with CRC present with metastatic diseases, while others develop metastasis later [4,5]. The liver is the primary site of metastasis, and liver metastasis is the leading cause of death in patients with CRC [5]. The median overall survival of untreated patients with colorectal liver metastases is only 5–20 months. Although a subset of patients achieve long-term remission following combined hepatic resection, 60–70% of patients still experience local or distant recurrence after surgery [2,6]. Colorectal liver metastasis (CRLM) is a critical focus, in which early detection and treatment are major challenges, and serves as a key factor in assessing patient prognosis.

Metabolic reprogramming of cancer cells is recognized as one of the hallmarks of cancer. These cancer cells utilize metabolites and energy to meet the demands of rapid tumor growth [6]. Previous studies on CRC have shown that amino acids (glutamine, glutamate, and histidine), fatty acids (oleic acid and stearic acid), and other metabolites (kynurenine and uric acid) are key features of metabolic dysregulation [7,8,9,10]. Diagnostic models comprising multiple metabolites have demonstrated excellent performance in disease diagnosis, prognostic assessment, and recurrence risk prediction [9,11]. Cancer metastasis is a multi-phase process in which primary tumor cells invade the surrounding stroma, disseminate through the circulation, and develop into metastatic lesions at distant sites, with metabolic reprogramming playing a pivotal role throughout this process. A previous review reported that the development and progression of CRLM are closely associated with metabolic syndrome [12]. This process is driven by regulating the metabolic reprogramming of tumor cells via multiple pathways involving lipids, glucose, and amino acids, as well as by remodeling the hepatic tumor microenvironment to form pre-metastatic niches.

Krishnan et al. specifically investigated the plasma lipid metabolites associated with CRC staging [13]. Using high-resolution liquid chromatography–mass spectrometry combined with machine learning models, they found significant differences in 16 lipid subclasses across different CRC stages and in patients with liver metastasis. Nevertheless, the plasma-based analysis in this study did not reveal the tissue origin or spatial distribution of these lipid changes; thus, many studies have been based on tissue homogenates or single-cell suspensions. For example, glucose metabolism intermediates, such as lactate, citrate, and succinate, were altered in CRC tissues, and temporal heterogeneity based on tumor tissue metabolism provided important evidence for distinguishing early- from late-stage CRC [14,15]. However, these studies disrupted the critical spatiotemporal heterogeneity information within tumors [16].

Advancements in mass spectrometry imaging (MSI) techniques have greatly advanced tumor heterogeneity visualization and contributed to spatially resolved metabolomics in cancer research [17]. For example, MSI and image segmentation have been used to assess tumor margins and the heterogeneity of CRC [18]. Recent spatially resolved metabolomics studies focusing on CRLM have made substantial progress using two MSI techniques: matrix-assisted laser desorption ionization (MALDI)-MSI and desorption electrospray ionization (DESI)-MSI. Through methodological optimization, such as the use of conductive tape or on-tissue derivatization to enhance metabolite detectability, these techniques have successfully mapped lipids, amino acids, and other metabolites in CRLM tissues [19,20,21]. Key findings from these studies highlight significant spatial metabolic heterogeneity in CRLM: specific lipids (e.g., phosphatidylinositols and sulfatides) and amino acids (e.g., glutamate and glutamine) are differentially enriched in metastatic foci versus adjacent liver tissue [19,20,21]; unsaturated/saturated sulfatide ratios correlate with disease-free survival in patients with CRLM [22]; and oxidative phosphorylation upregulation emerges as a critical metabolic reprogramming event promoting liver metastasis [6]. Moreover, MSI-derived metabolic signatures have been used to construct objective pathological response scores that correlate with patient prognosis, thereby overcoming the subjectivity of traditional histopathological assessment [23]. The identification of key dysregulated metabolites in situ can offer a comprehensive view of the molecular alterations driving metastasis and potentially uncover novel biomarkers or therapeutic targets [24,25,26]. Despite these advances, existing MSI-based methods in CRLM still have limitations; most single detections are restricted to a small number of metabolite classes, mainly focusing on lipids, amino acids, or carbohydrates, and on-tissue derivatization is sometimes required. Therefore, research to simultaneously detect multiple classes of metabolites in a single run is lacking. Overall, MSI allows detailed mapping of metabolites within heterogeneous tumor tissues, shedding light on metabolic reprogramming in complex tissue structures.

In summary, this study aimed to investigate the metabolic landscape and identify candidate metabolites and the underlying metabolic regulatory alterations in mCRC based on spatially resolved metabolomics. MSI technologies were applied to map the metabolic profiles of normal colorectum, primary tumor, liver metastasis, and normal liver tissue regions from patients with CRC, with and without liver metastasis. By integrating spatially resolved metabolomic data with hematoxylin and eosin (H&E) staining, we characterized metabolic reprogramming within distinct regions of heterogeneous tumor tissues in CRLM. Alterations in glycerolipid, acylcarnitine, and amino acid metabolism during tumor progression were further dissected. These findings provide potential tissue-specific metabolic signatures and targets for the diagnosis and treatment of colorectal liver metastasis.

## 2. Materials and Methods

### 2.1. Experimental Design and Subject Information

Patients diagnosed with CRC who provided written informed consent were prospectively enrolled. A total of ten patients who underwent radical or synchronous resection of the primary colorectal tumor and liver metastases at Peking Union Medical College Hospital were included. The inclusion criteria were as follows: histopathologically confirmed colorectal carcinoma and metastasis-free CRC or CRLM. Patients were excluded if the quantity or quality of the surgically resected tissue samples was insufficient to perform subsequent experiments. The clinicopathological characteristics of the patients, including age, gender, tumor histological type, T stage, American Joint Committee on Cancer (AJCC) stage, and lymph node involvement status, are summarized in Table S1. The disease extent was comprehensively evaluated using chest and abdominal computed tomography and rectal and liver magnetic resonance imaging, as appropriate. The application to access patient tissue samples was approved by the Ethics Committee of the Chinese Academy of Medical Sciences & Peking Union Medical College, Peking Union Medical College Hospital (ethical approval document number: I-25PJ0484). All procedures involving human participants were conducted in strict accordance with relevant ethical regulations and guidelines for human research.

### 2.2. Biospecimen Collection and Processing

The surgically resected tissues were collected, immediately placed in dry ice and then transferred to a −80 °C refrigerator. Areas with necrosis or hemorrhage were avoided. In total, ten normal colorectum tissues, ten colorectal tumor tissues, and three liver metastasis tissues were collected from ten patients with CRC. Then, the tissues were embedded in optimal cutting temperature (OCT) compound and cut into 12 μm frozen sections and 7 μm frozen sections at −20 °C on a cryostat microtome (Leica CM 1860 UV, Wetzlar, Germany). These tissue sections were mounted onto SUPERFROST PLUS slides (Thermo Scientific, San Jose, CA, USA). The 12 μm tissue sections were used for airflow-assisted desorption electrospray ionization (AFADESI)-MSI in positive ion mode. The 7 μm sections were used for hematoxylin–eosin staining. The tissue samples were mixed with grinding steel beads and 500 μL of 2:2:1 ACN/MeOH/H_2_O (*v*/*v*/*v*) as the extract solution. The mixture was vortexed, homogenized, and sonicated. The samples were then incubated to precipitate the proteins and centrifuged. The supernatant was used for LC–HRMS/MS analysis.

### 2.3. Hematoxylin and Eosin Staining

Frozen tissue sections were fixed in 95% ethanol for 10–30 s and briefly rinsed in running water for 1–2 s. Subsequently, the sections were stained with a hematoxylin solution for 60 s, followed by rinsing in running water to remove excess hematoxylin solution for 5–10 s. The sections were then immersed in a 1% hydrochloric acid ethanol solution for differentiation for 1–3 s, briefly rinsed in running water for 10–20 s, and subsequently placed in a bluing solution (1% ammonia water) for 5 s, followed by flushing with running water for 10 s. Counterstaining was performed with a 0.5% eosin solution for 20–30 s, and the sections were flushed with running water for 30 s. Dehydration was conducted sequentially in an ethanol gradient: immersion in 80% ethanol for 10–20 s, 95% ethanol for 10–20 s, absolute ethanol I for 20 s, absolute ethanol II for 5–10 s, and absolute ethanol III for 30 s. Thereafter, the sections were rendered transparent by immersion in clearing solution I for 30 s and clearing solution II for 30 s, followed by wet mounting with a mounting medium. The H&E-stained frozen sections were scanned using a NanoZoomer 1 Digital Slide Scanner (Hamamatsu Photonics K.K., Hamamatsu, Japan), and images were acquired and viewed using NDP.view2 software (Hamamatsu Photonics K.K., Hamamatsu, Japan).

### 2.4. MSI and LC-MS/MS Analysis

AFADESI-MSI [27] setup coupled with an Orbitrap Exploris 240 mass spectrometer (Orbitrap MS, Thermo Scientific) was used to perform the tissue section MSI. The AFADESI-MSI setup conditions were set as follows: horizontal speed, 0.20 mm/s; vertical step size, 0.2 mm; and spraying solvent, 9:1 ACN/H_2_O (*v*/*v*), with a flow rate of 5 μL/min. The mass spectrometric parameters were set as follows: scan mode, full MS within the range of *m*/*z* 70–1000; spray voltage, 4.5 kV (positive); capillary temperature, 350 °C; and resolution, 90,000. LC–MS/MS analysis was performed on a Vanquish UHPLC system (Thermo Scientific) with a Waters BEH Amide column (2.1 mm × 50 mm, 1.7 μm) coupled to an Orbitrap Exploris 120 mass spectrometer (Thermo Scientific) used for metabolite identification. The MS data were acquired using full MS and ddMS^2^, collision energy, and SNCE 20/30/40.

### 2.5. MSI and LC-MS/MS Data Processing

Raw MSI data files collected from each tissue section were converted into serial cdf format files using Xcalibur 4.0 (Thermo Scientific, San Jose, CA, USA). The files were then imported into MassImager (MassImager 1.0, Beijing, China) [28] software to reconstruct the MS images and extract the average mass spectra of any regions of interest. Peak alignments and normalization were based on MarkerView™ software 1.2.1 (AB Sciex, Toronto, ON, Canada). Mass shift evaluation and metabolite annotation were based on self-built databases and our established Python (version 3.12.7) scripts MSIDAT (https://github.com/Yingzhu96/MSIDAT, accessed on 12 April 2025). The mass accuracy was set at 5 ppm, allowing [M + H]^+^, [M + Na]^+^, [M + K]^+^, [M + NH_4_]^+^, [M − H_2_O + H]^+^, and [M]^+^ as positive adducts. Metabolite identification was performed using the R package (version 4.4.1) based on the LC-MS/MS spectra, which were compared with the MS/MS spectra of standard compounds and public databases, including HMDB (http://www.hmdb.ca, accessed on 2 December 2024), METLIN (https://metlin.scripps.edu, accessed on 2 December 2024), MoNA (https://mona.fiehnlab.ucdavis.edu, accessed on 2 December 2024), and PubChem (https://pubchem.ncbi.nlm.nih.gov, accessed on 2 December 2024).

### 2.6. Statistical Analysis and Metabolic Network Analysis

SIMCA-P (Umetrics AB) was used for principal component analysis (PCA) and orthogonal partial least squares discriminant analysis (OPLS-DA). Univariate statistical analysis was based on self-written MATLAB (version R2024b) scripts as described previously [29], and the *p* values were corrected with Benjamini−Hochberg false discovery rates (FDRs). Statistically altered ions were set as FDR-corrected *p* value < 0.05 and |log2 fold change| > 0.25. The compound names were input into the pathway analysis models of MetaboAnalyst 6.0 [30]. Homo sapiens in the Kyoto Encyclopedia of Genes and Genomes (KEGG) was selected as the reference database, and the *p* value cutoff was set at 0.05. Venn diagrams and volcano plots were obtained from Hiplot Pro (https://hiplot.com.cn/, accessed on 13 January 2026), and the Sankey diagram and horizontal lollipop plot were generated by https://www.bioinformatics.com.cn/ (accessed on 14 January 2026). Receiver operating characteristic (ROC) curve analysis was performed using SPSS Statistics (version 27.0) and GraphPad Prism software (version 10.1.2). The area under the curve (AUC) was used to measure the strength of the discriminators. The Python (version 3.12.7) scikit-learn package was used for the 5-fold cross-validation.

## 3. Results

### 3.1. Comprehensive Metabolomic Landscape Mapping of Colorectal Cancer

**Figure 1:**
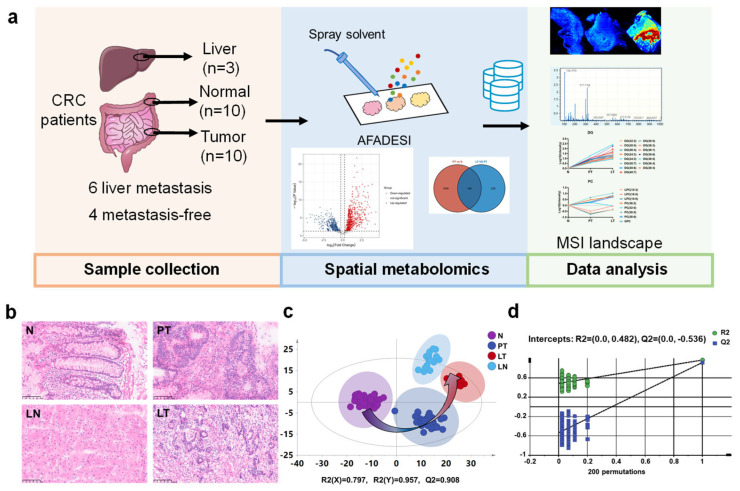
Comprehensive metabolomic landscape mapping of colorectal cancer. (**a**) Experimental design and data analysis workflow of this study. (**b**) Hematoxylin–eosin staining of N, PT, LT, and LN with a scale bar of 100 μm. (**c**) OPLS-DA plots of AFADESI-MSI data in N, PT, LT, and LN. (**d**) Results of the permutation test for OPLS-DA. N: normal colorectal mucosal region, PT: primary tumor region, LT: liver tumor region, LN: normal liver tissue region.

To explore the metabolome landscape of CRC, paired normal colorectal and colorectal tumor samples from four patients with metastasis-free CRC were collected. Normal colorectal, colorectal tumor, and liver metastasis tissue samples from six patients with liver metastasis CRC were collected. Liver metastasis samples were obtained from three of these six patients, resulting in a total of twenty-three tissue samples analyzed by metabolomics (Figure 1a). Patients with pathological or imaging evidence of liver metastasis at the time of CRC diagnosis were defined as patients with metastatic CRC, while patients without liver or other distant organ metastasis confirmed by postoperative pathology were defined as patients with metastasis-free CRC. Clinical information of the included patients is summarized in Table S1.

H&E staining of normal colorectal, primary tumor, and liver metastatic tumor samples reflected the heterogeneous tissue structure, and all H&E-stained slides were checked by pathological experts, validating the accuracy of our sampling. The normal colorectal mucosal region (N), primary tumor region (PT), liver tumor region (LT), and normal liver tissue region (LN) were selected as the regions of interest (ROIs) in the following study (Figure 1b). Adjacent frozen tissue sections were analyzed using the AFADESI-MSI platform and preprocessed using MassImager software by overlaying pathologist-annotated H&E-stained regions with the MSI images. Next, OPLS-DA was applied to analyze the MSI data of the twenty-three tissues. Spatially resolved metabolome data of the normal colorectal mucosal, primary tumor, liver tumor, and liver normal tissue regions were clustered separately (Figure 1c). The 200 permutation tests showed that Q^2^ and R^2^ values of the permuted models were consistently lower than those of the original model, and the Q^2^ regression line intercept was −0.536, suggesting no overfitting of the OPLS-DA model (Figure 1d). In the MSI data, the metabolomic profile was ordered as follows: normal colorectal mucosal, primary tumor, liver tumor, and normal liver tissue regions. The data of adjacent cell types were more similar than those of nonadjacent cell types, which is consistent with the developmental process of cancer cells.

### 3.2. Tumor-Associated Metabolic Reprogramming in Colorectal Tissues

**Figure 2:**
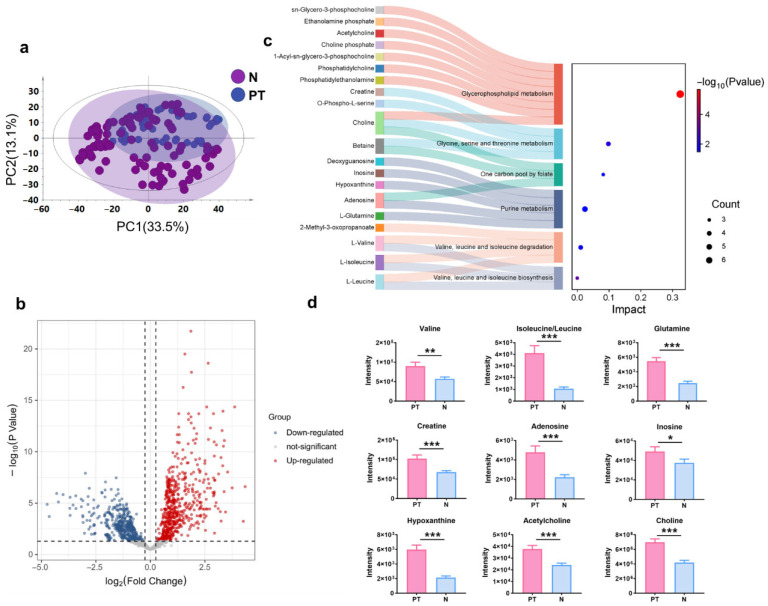
Tumor-associated metabolic reprogramming in colorectal tissues. (**a**) PCA plots of AFADESI-MSI data in N and PT. (**b**) Differentially expressed ions in PT vs. N. Red dots: significantly upregulated ions in PT; blue dots: significantly downregulated ions in PT. (**c**) Pathway analysis of significantly altered metabolites (*p* < 0.05). (**d**) Relative quantitation of significantly different metabolites between N and PT (means ± SEM). FDR-corrected, * *p* < 0.05, ** *p* < 0.01, and *** *p* < 0.001. N: normal colorectal mucosal region, PT: primary tumor region.

To investigate the metabolome profile of colorectal tissues, MSI features were extracted from ROIs selected from the normal colorectal mucosal and primary tumor regions. A PCA model based on these AFADESI-MSI data distinguished between normal mucosa and the tumor (Figure 2a). Univariate statistical analysis was performed to screen for differential ions, identifying 1174 ions that were statistically altered (FDR-corrected *p* value < 0.05, |log2 fold change| > 0.25), with 590 and 584 ions significantly upregulated and downregulated, respectively, as shown in Figure 2b. These ions were further annotated using the MSIDAT tool and self-built databases, and the identified differential metabolites included amino acids, purines, cholines, and glyceryl phosphatide species. The discriminating metabolites were then imported into MetaboAnalyst 6.0 using the KEGG database to perform metabolic pathway matching analysis. This analysis suggested that six pathways were significantly altered with *p* < 0.05, including glycerophospholipid metabolism; valine, leucine and isoleucine biosynthesis; glycine, serine and threonine metabolism; valine, leucine and isoleucine degradation; one carbon pool by folate; and purine metabolism (Figure 2c and Table S2). Subsequent analysis revealed that the amino acid pathways (including valine, leucine/isoleucine, and glutamine), purine metabolism pathway (involving creatine, adenosine, inosine, and hypoxanthine), choline, and acetylcholine were significantly upregulated in the primary tumor region compared to the normal mucosal region (Figure 2d). Figure S1 shows representative MS/MS spectra of the identified metabolites.

### 3.3. Tumor Metabolic Reprogramming in Liver Metastasis Tissues

**Figure 3:**
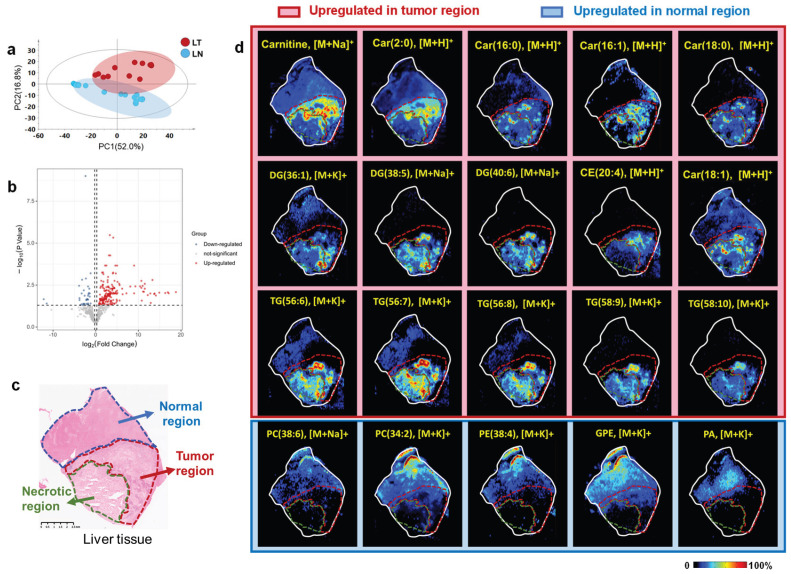
Tumor metabolic reprogramming in liver metastasis tissues. (**a**) PCA score plots of AFADESI-MSI data in LT and LN. (**b**) Differentially expressed ions in LT vs. LN. Red dots: significantly upregulated ions in LT; blue dots: significantly downregulated ions in LT. (**c**) Hematoxylin–eosin staining of normal region, tumor region, and necrotic region in the liver tissue, with a scale bar of 2.5 mm. (**d**) Representative MS images of regulated lipids and acylcarnitine metabolites in liver tissue section. LT: liver tumor region, LN: normal liver tissue region.

To further investigate the metabolome profile of normal and tumor regions in liver tissues, MSI data from ROIs in the liver tumor and adjacent normal liver tissue regions were extracted, and a PCA model clearly distinguished between tumor and normal liver tissues (Figure 3a). Univariate statistical analysis identified 219 ions that were significantly altered, of which 183 and 36 were upregulated and downregulated, respectively (Figure 3b). After putative annotation of these differential ions, multiple lipids, including triglycerides (TGs), diglycerides (DGs), and cholesteryl esters (CEs), were found to be significantly upregulated in metastatic liver tumor tissues. As shown in Figure 3d, TG (56:6), TG (56:7), TG (56:8), TG (58:9), TG (58:10), DG (36:1), DG (38:5), DG (40:6), and CE (20:4) were enriched in tumor and/or necrotic regions relative to normal region. In addition, acylcarnitine species accumulated in the tumor region, including carnitine (Car), Car (2:0), Car (16:0), Car (16:1), Car (18:0), and Car (18:1). Given the central role of the liver in lipid metabolism and the critical function of acylcarnitines in mitochondrial β-oxidation, these metabolic patterns may suggest an enhancement of lipid synthesis, impaired fatty acid oxidation, and/or mitochondrial dysfunction in the liver tumor region. In contrast, the glycerophospholipid species were significantly downregulated, including phosphatidylcholine (PC) 38:6, PC (34:2), phosphatidylethanolamine (PE) 38:4, glycerophosphatidylethanolamine (GPE), and phosphatidic acid (PA).

### 3.4. Metabolic Changes During Tumorigenesis and Metastasis in Colorectal Cancer

**Figure 4:**
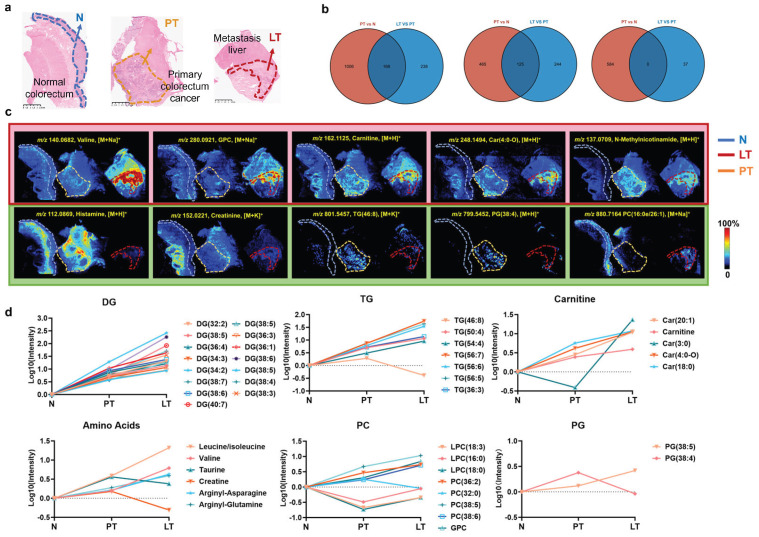
Metabolic changes during tumorigenesis and metastasis in colorectal cancer. (**a**) Hematoxylin–eosin staining of normal colorectal, primary tumor, and liver tumor tissues, and N, PT, LT region, with a scale bar of 2.5 mm. (**b**) Venn diagram of significantly differential ions among different comparison groups. (**c**) Representative MS images of changed metabolites in liver tissue section. (**d**) The average levels of the representative metabolites change across N, PT and TT. The intensities were normalized to the intensities of N. N: normal colorectal mucosal region, PT: primary tumor region, LT: liver tumor region.

Based on H&E staining, normal colorectal mucosal regions were precisely selected from normal colorectal tissues and primary tumor tissues containing normal mucosal areas, primary tumor regions from primary tumor tissues, and liver tumor regions from liver metastasis tissues (Figure 4a). Subsequent analyses identified common and specific changes across the normal colorectal mucosal, primary tumor, and liver tumor regions. First, a PCA model clearly separated the liver tumor and primary tumor regions (Figure S2a), and univariate statistical analysis identified 406 ions that were significantly altered, with 369 and 37 ions upregulated and downregulated, respectively (Figure S2b). Subsequently, significant differential ions from the comparisons of primary tumors vs. normal tissues and metastatic tumors vs. primary tumors were integrated and analyzed. Figure 4b shows that 1412 ions were significantly changed in at least one of the pairwise comparisons, and 168 common ions were significantly changed in both of the pairwise comparisons of the three different tissue types. Among them, 125 ions were upregulated in both comparisons, and no ions were downregulated in both comparisons. To explore the functional roles of the expressed ions in CRC tumorigenesis and metastasis, these common ions were annotated to their corresponding metabolites using MSIDAT, and distinct patterns of upregulation and downregulation were observed among the identified metabolites. For example, Figure 4c shows that valine, glycerophosphocholine (GPC), carnitine, Car (4:0-O), and N-methylnicotinamide significantly increased across N, PT, and LT; in contrast, histamine, creatinine, TG (46:8), and PG (38:4) increased from N to PT but decreased from PT to LT. In addition, PC (16:0e/26:1) increased from PT to LT but decreased from N to PT. Classifying these metabolites according to their types, all DG species steadily increased across N, PT and LT, and most TG species also steadily increased across N, PT and LT, except for TG (46:8). In addition, most carnitine species significantly increased as well. The upregulation of these lipids indicates enhanced lipid synthesis, impaired fatty acid oxidation, and/or mitochondrial dysfunction during tumorigenesis and metastasis. Nevertheless, amino acid species exhibited complex changes: histamine, creatinine, and taurine levels decreased, whereas valine and leucine/isoleucine levels steadily increased (Figure 4d). In agreement with a previous study, targeted metabolomics showed increased levels of valine and leucine/isoleucine in tumor tissues compared with those in adjacent normal tissues in patients with colon cancer [31]. In addition, PC and phosphatidylglycerol (PG) showed complex changes. The changes in these metabolites in different tissues reflect the complexity of metabolite regulatory networks related to cancer progression.

### 3.5. Metabolic Signatures and Multivariate Models to Discriminate and Characterize Metastatic and Non-Metastatic CRC

**Figure 5:**
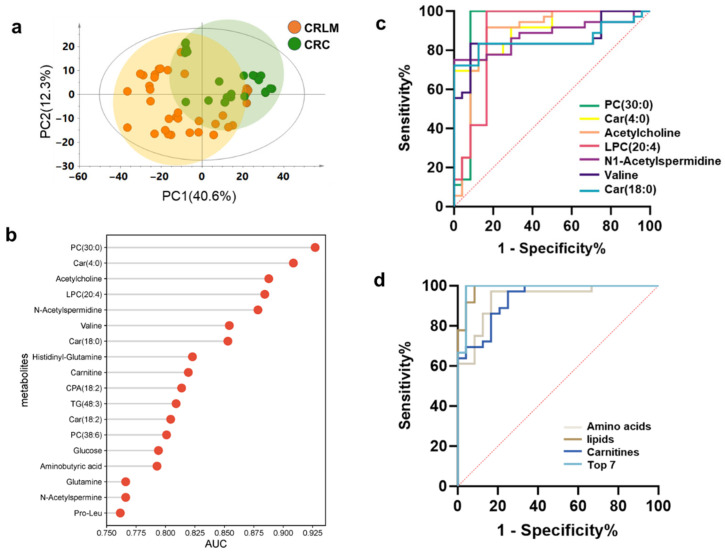
Metabolic markers and multivariate models to discriminate and characterize metastatic and non-metastatic CRC. (**a**) PCA score plots of AFADESI-MSI data in CRC and CRLM. (**b**) Receiver operating characteristic curve analysis for potential metabolic signatures to distinguish CRLM and CRC, scatter plot showing 18 metabolites had values of AUC > 0.75. (**c**) AUC plots for the top seven potential metabolic signatures. (**d**) AUC plots for the multivariate models. AUC, area under the curve.

CRLM typically has a poorer prognosis than non-metastatic CRC, and the identification of exploratory diagnostic signatures and therapeutic targets for CRLM is of great clinical significance. Metabolite expression patterns in primary tumors were compared between CRC patients with and without liver metastasis to explore potential metabolic drivers of cancer metastasis. Figure 5a shows that the MSI data of CRLM were distinct from those of metastasis-free CRC, and univariate statistical analysis showed 689 differential ions. After annotating these ions with MSIDAT and visualizing them in MS images, 122 distinct metabolite ions were identified, with the majority being downregulated in CRLM compared with non-metastatic CRC. Pathway analysis was then performed for differential metabolites based on the KEGG database and eight remarkably altered pathways were identified with *p* < 0.05, including glycerophospholipid metabolism; galactose metabolism; glycine, serine, and threonine metabolism; one carbon pool by folate; alanine, aspartate, and glutamate metabolism; butanoate metabolism; arginine and proline metabolism; and neomycin, kanamycin, and gentamicin biosynthesis (Table S3). Univariate ROC curve analysis was performed for the 122 differential metabolite ions to identify potential metabolic signatures capable of distinguishing CRLM from non-metastatic CRC. A total of 18 metabolites had a high (AUC ≥ 0.75) group-specific discriminative potential (Figure 5b). The top seven metabolites (AUC ≥ 0.85) are shown in Figure 5c, including PC (30:0), Car (4:0), acetylcholine, LPC (20:4), *N*-acetylspermidine, valine, and Car (18:0), highlighting their potential to distinguish CRLM from metastasis-free CRC based on primary tumor tissues. The ROC curve analysis of individual features is limited by its disregard for the interrelationships among features, and multivariate ROC curve analysis was subsequently performed using metabolites of the same class or those with high AUC values. Four amino acids (*N*-acetylspermidine, valine, glutamine, and *N*-acetylspermine), four lipids (PC (30:0), LPC (20:4), TG (48:3), and PC (38:6)), four carnitines (Car (4:0), Car (18:0), carnitine, and Car (18:2)), and the top seven AUC metabolites were used for multivariate models. Their AUC values were all greater than 0.9, indicating that these models were good discriminators. The four-lipid multivariate model was identified as the most effective panel with a higher diagnostic efficiency (AUC = 0.987) with binary logistic regression, and 5-fold cross-validation AUC was 0.967 ± 0.044, indicating good robustness and generalizability. This underscores the high sensitivity and specificity of these lipids for CRLM and their great potential as spatially resolved clinic metabolic signatures.

## 4. Discussion

Compared with previous MSI studies on CRC, this study simultaneously profiled multiple metabolite classes and characterized the spatial metabolic trajectory of CRLM. This study was performed on a total of 23 normal mucosal, primary tumor, liver tumor, and normal liver samples. By integrating pathological staining for ROI selection, the metabolomic landscape was mapped and in situ cancer metabolic reprogramming was revealed. A previous study on CRLM based on a three-dimensional genome and RNA-seq data showed that adjacent cell types are more similar and liver metastasis is more similar to the normal liver than to other tissue types [32]. In the metabolome, this study found similar characteristics in that the metabolic profile of liver metastasis was more similar to that of the normal liver than to other tissue types. In accordance with prior reports on RNA-seq data [32], the metabolomic profiles were sorted according to the order of normal colorectal mucosa, primary tumor, liver tumor, and normal liver tissues.

Some studies on the metabolomics of CRC have revealed lipids (including GPC, phosphatidylinositols, and sulfatides), amino acids (including branched-chain amino acids (BCAAs) and glutamine), and glucose metabolism shifts throughout the tumorigenic and metastatic states [13,33,34,35]. Spatially resolved metabolomics further revealed the regional metabolic reprogramming of tumor tissues and metastatic foci [36]. In this study, the analysis of tumor-associated metabolic features in colorectal tissues identified significant alterations in the glycerophospholipid metabolism; valine, leucine, and isoleucine biosynthesis; glycine, serine, and threonine metabolism; valine, leucine, and isoleucine degradation; one carbon pool by folate; and purine metabolism pathways. Previous studies have reported that BCAAs were expressed at higher levels in colon tumor tissues than in normal tissues [31], and valine has been previously reported to be a metabolic biomarker of CRC risk [37]. Elevated BCAA levels in tissues are likely to provide nutrients to support tumor cell growth. Further investigation of liver tumor-associated metabolic alterations identified and visualized a multitude of region-specific lipids in the liver tumor region. Upregulation in Car, TG, DG, and CE species was obvious, indicating active lipid synthesis, incomplete oxidation of fatty acids, and/or mitochondrial dysfunction of the liver. This finding aligns with prior studies that reported that the contents of many TG species, such as TG (56:5) and TG (56:6), were elevated in the tumor tissue, and key enzymes for new fat synthesis significantly increased in tumors [38]. These metabolic alterations are consistent with research findings, demonstrating that mitochondrial fatty acid oxidation supports metastatic adaptation by providing ATP and NADPH under nutritional stress [39]. Mitochondrial metabolic reprogramming has been proposed to sustain lipid catabolism within the metastatic microenvironment of CRLM [40].

Cancer metastasis is a major cause of cancer recurrence, lethality, and a poor prognosis. This MSI-based study identified distinct metabolomic features between LT and PT and further delineated the spatial patterns of metabolite reprogramming across progression. For example, valine, GPC, carnitine, Car (4:0-O), and *N*-methylnicotinamide steadily increased, while histamine, creatinine, TG (46:8), PG (38:4), and PC (16:0e/26:1) showed intricate changes across N, PT, and LT. The upregulation and downregulation patterns of these metabolites differ from one another. The results demonstrated that the metabolism of glycerides, acylcarnitines, amino acids, and phosphatidylcholine collectively reflects the metabolic reprogramming associated with CRLM progression. In addition to the changes within the tumor, new evidence indicates that interactions between tumor cells and hepatocytes, hepatic stellate cells, and immune cells in the liver microenvironment further promote metabolic adaptation and immune evasion during CRLM [41,42]. Metabolic reprogramming of CRC and its liver metastasis has formed several universal metabolic themes, including changes in glucose metabolism (oxidative phosphorylation vs. glycolysis) [6], amino acid pathways [19], and lipid pathways [43]. These metabolic changes are closely related to the rapid proliferation of tumor cells and adaptive survival of metastatic cells in the liver microenvironment. However, no universal metabolomic signatures for CRC liver metastasis have been agreed upon across all studies. The main reasons for this include differences in study populations, sample processing methods, detection technologies, and analytical strategies. This study provided another spatial metabolomic perspective for the screening of discriminative metabolic signatures, and the consistency of lipid and BCAA metabolic disorders found in this and previous studies suggests that lipid and amino acid metabolism may be a potential universal target for CRC liver metastasis diagnosis and treatment. In addition, the multivariate model comprising four lipids was deemed an effective panel, underscoring their sensitivity to CRLM and potential as discriminative features. Although the AUC was high, further validation is required.

In addition, differences in tissue genome characteristics across patients that are metastasis-free and those with metastasis have been reported. Future research on CRLM metabolic signatures will need to integrate multidimensional analyses of both blood and tissue samples. Combining metabolic characteristics with genomic information is expected to provide a more comprehensive basis for the precise diagnosis of CRLM, along with considering tumor spatial heterogeneity as a key variable to construct diagnostic and prognostic models with greater clinical efficacy.

The relatively small sample size, particularly for liver metastasis tissues, may limit the statistical power and generalizability of the findings. The results are experimental and require further validation in larger, independent cohorts. Future studies with a larger expanded sample size and a more comprehensive set of clinical and pathological parameters (including tumor molecular subtypes and genomic status) are required to identify additional differential metabolomic signatures [44,45]. For example, given the variation between left and right colon cancers in terms of clinical parameters including treatment response [46,47], distinct metabolomic profiles exist in seven CRC subsites (cecum, ascending colon, transverse colon, descending colon, sigmoid colon, rectosigmoid colon, and rectum) based on tissue homogenates. Therefore, future cohorts should ideally incorporate a larger number of patients and stratify sample subsites. Furthermore, we minimized OCT usage to reduce polyethylene glycol-induced ion suppression [48]; however, matrix interference was not fully avoided, and the influence of OCT on ion intensity in MSI analysis was a limitation. In addition, the spatial resolution of AFADESI-MSI also hinders metabolic characterization at the cellular and subcellular levels, necessitating future efforts to enhance spatial resolution by decreasing the X and Y step rates and reducing the spray spot size. The observed metabolic differences may be predominantly driven by cancer progression and may also be influenced by microenvironmental factors. Additionally, further metabolite identification via in situ tandem MS and/or ion mobility is recommended to fully elucidate the underlying molecular mechanisms and more precisely characterize complex metabolites and lipids.

## 5. Conclusions

In this study, CRC patients with and without liver metastasis were enrolled to comprehensively map the metabolite landscape across matched normal colorectal, primary tumor, and metastatic liver tissues. The altered metabolites and metabolic pathways involved in cancer metastasis were investigated using spatially resolved metabolomics. Clustering analysis of metabolite expression across normal mucosal, primary tumor, liver tumor, and normal liver regions revealed continuous alterations in metabolite expression patterns. Distinct metabolic alterations in DG, TG, acylcarnitine, and amino acid levels were identified during the progression from the normal colorectal mucosal region to the primary tumor region and then to liver metastasis. Finally, a four-lipid multivariate panel was constructed to distinguish between CRLM and metastasis-free CRC. Future studies with larger cohorts and multi-center validation are needed to confirm these findings.

## Data Availability

The datasets in the study are available from the corresponding author on reasonable request.

## Supplementary Materials

The following supporting information can be downloaded at https://www.mdpi.com/article/10.3390/metabo16050293/s1, Figure S1: MS/MS spectra of the identified valine, leucine/isoleucine, glutamine, creatine, adenosine, inosine, hypoxanthine, acetylcholine, choline; Figure S2: Metabolic profile differences between PT and LT; Table S1: Clinical data and pathological characteristics of the patients; Table S2: Pathway analysis of significantly altered metabolites between normal colorectum and colorectal tumor tissues; Table S3: Pathway analysis of significantly altered metabolites between CRLM and CRC without metastasis.
