# Supplementary material for: Spatially Resolved Metabolomic Profiling Reveals Progression-Associated Metabolic Reprogramming in Colorectal Liver Metastasis
Source: Metabolites. 2026 Apr 24;16(5):293. doi: 10.3390/metabo16050293 (PMC13208894; doi:10.3390/metabo16050293)
Supplement: Supplementary file 1 [file metabolites-16-00293-s001.zip › metabolites-4249600-supplementary.pdf]

Supporting Information

Spatially Resolved Metabolomic Profiling Reveals Progression-Associated Metabolic Reprogramming in Colorectal Liver Metastasis

Ying Zhu [1 †], Yixuan Cai [1 †], Qianyu Wang [2], Hanchuan Guo [2], Qianqian Xie [1], Yingshi Xiang [2], Songlin Yu [1], Bin Wu [2,*] and Ling Qiu [1, 3,*]

[1] Department of Laboratory Medicine; Peking Union Medical College Hospital, Chinese Academy of Medical Sciences & Peking Union Medical College, Beijing 100730, China

[2] Department of General Surgery, Peking Union Medical College Hospital, Chinese Academy of Medical Sciences & Peking Union Medical College, Beijing, 100730, China

[3] State Key Laboratory of Complex Severe and Rare Diseases, Peking Union Medical College Hospital, Chinese Academy of Medical Sciences & Peking Union Medical College, Beijing 100730, China

†Y.Z. and Y.C. contributed equally to this work

*Corresponding author.

Email: qiul@pumch.cn (L.Q.), wubin@pumch.cn (B.W.)

Valine

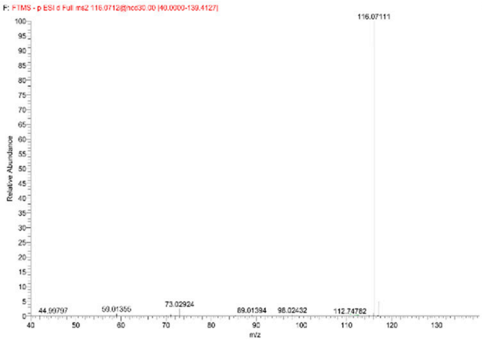

Leucine/Isoleucine

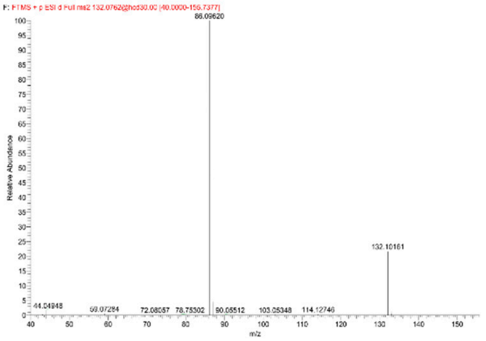

Glutamine

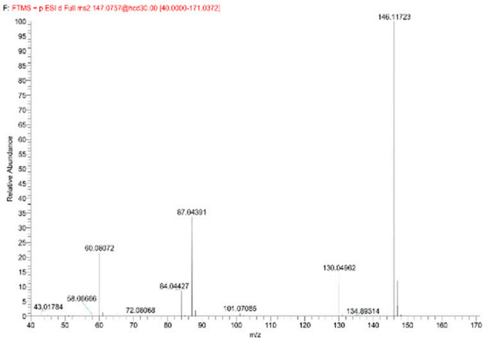

Creatine

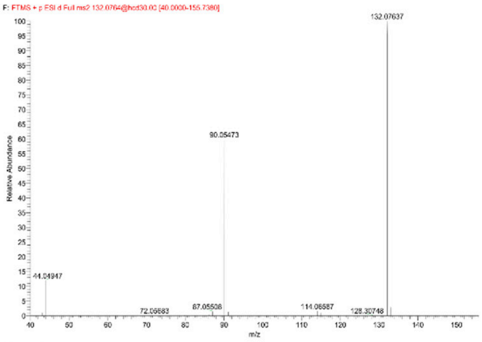

Adenosine

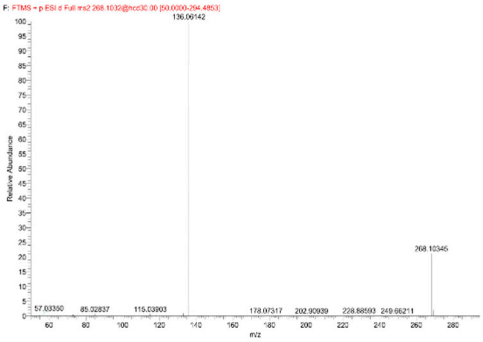

Inosine

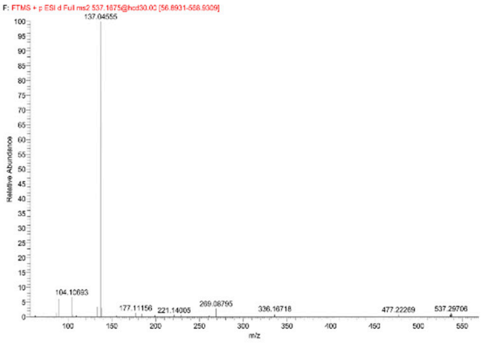

Hypoxanthine

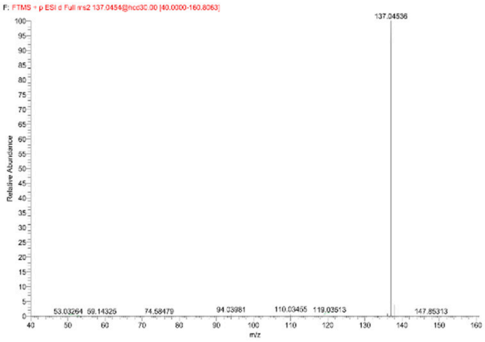

Acetylcholine

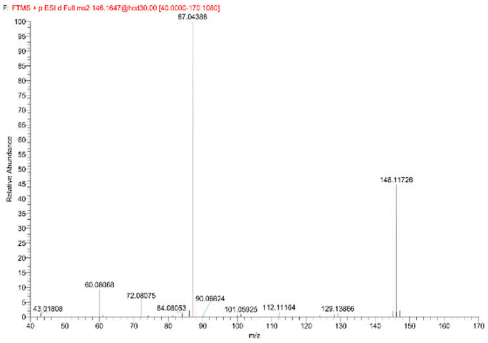

Choline

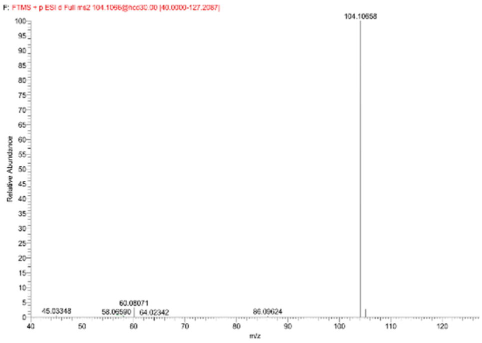

Figure S1. MS/MS spectra of the identified valine, leucine/isoleucine, glutamine, creatine, adenosine, inosine, hypoxanthine, acetylcholine, choline.

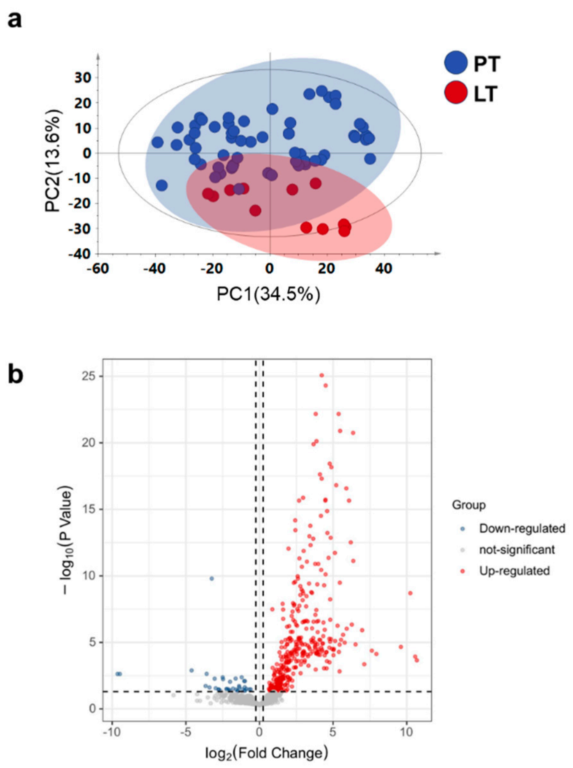

Figure S2. Metabolic profile differences between PT and LT. (a) PCA plots of AFADESI-MSI data in PT and LT. (b) Differentially expressed ions in LT vs. PT. Red dots: significantly upregulated ions in the LT, blue dots: significantly downregulated ions in the LT. PT: primary tumor region, LT: liver tumor region.

Table S1. Clinical data and pathological characteristics of the patients.

	Number	%
**Total number**	10	
**Age**, Mean±SD	61±8.47	

**Gender**		
Male	8	80
Female	2	20
**Metastasis**		
Liver metastasis	6	60
Metastasis-free	4	40
**Pathology**		
Adenocarcinoma	9	90
Mucinous carcinoma	1	10
**Differentiation**		
Well differentiated	0	0
Moderately differentiated	10	100
Poorly differentiated	0	0
**T-Stage**		
T1	0	0
T2	2	20
T3	7	70
T4	1	10
**AJCC Stage**		
I	2	20
II	1	10
III	1	40
IV	6	60

**Lymph node status**		
N0	5	50
N1	2	20
N2	3	30

Table S2. Pathway analysis of significantly altered metabolites between normal colorectum and colorectal tumor tissues.

Pathway	*P*	Impact
Glycerophospholipid metabolism	2.07E-06	0.32418
Valine, leucine and isoleucine biosynthesis	0.000873	0
Glycine, serine and threonine metabolism	0.009833	0.09802
Valine, leucine and isoleucine degradation	0.019213	0.01084
One carbon pool by folate	0.029116	0.08187
Purine metabolism	0.034108	0.02433

Table S3. Pathway analysis of significantly altered metabolites between CRLM and CRC without metastasis.

Pathway	*P*	Impact
Glycerophospholipid metabolism	7.39E-05	0.29057
Galactose metabolism	0.00018921	0.39113
Glycine, serine and threonine metabolism	0.00050723	0.12575
One carbon pool by folate	0.0018609	0.1572
Alanine, aspartate and glutamate metabolism	0.020252	0.2484
Butanoate metabolism	0.039074	0.0635
Arginine and proline metabolism	0.039304	0.08953
Neomycin, kanamycin and gentamicin	0.042271	0
